# Follicular Dendritic Cells of Lymph Nodes as Human Immunodeficiency Virus/Simian Immunodeficiency Virus Reservoirs and Insights on Cervical Lymph Node

**DOI:** 10.3389/fimmu.2018.00805

**Published:** 2018-04-19

**Authors:** Rajnish S. Dave, Pooja Jain, Siddappa N. Byrareddy

**Affiliations:** ^1^Department of Pharmacology and Experimental Neuroscience, University of Nebraska Medical Center, Omaha, NE, United States; ^2^Department of Microbiology and Immunology, Institute for Molecular Medicine and Infectious Disease, Drexel University College of Medicine, Philadelphia, PA, United States

**Keywords:** cervical lymph nodes, follicular dendritic cells, T follicular helper cells, central nervous system, human immunodeficiency virus, simian immunodeficiency virus, viral reservoirs, combined antiretroviral therapy

## Abstract

A hallmark feature of follicular dendritic cells (FDCs) within the lymph nodes (LNs) is their ability to retain antigens and virions for a prolonged duration. FDCs in the cervical lymph nodes (CLNs) are particularly relevant in elucidating human immunodeficiency virus (HIV)-1 infection within the cerebrospinal fluid (CSF) draining LNs of the central nervous system. The FDC viral reservoir in both peripheral LN and CLN, like the other HIV reservoirs, contribute to both low-level viremia and viral resurgence upon cessation or failure of combined antiretroviral therapy (cART). Besides prolonged virion retention on FDCs in LNs and CLNs, the suboptimal penetration of cART at these anatomical sites is another factor contributing to establishing and maintaining this viral reservoir. Unlike the FDCs within the peripheral LNs, the CLN FDCs have only recently garnered attention. This interest in CLN FDCs has been driven by detailed characterization of the meningeal lymphatic system. As the CSF drains through the meningeal lymphatics and nasal lymphatics *via* the cribriform plate, CLN FDCs may acquire HIV after capturing them from T cells, antigen-presenting cells, or cell-free virions. In addition, CD4+ T follicular helper cells within the CLNs are productively infected as a result of acquiring the virus from the FDCs. In this review, we outline the underlying mechanisms of viral accumulation on CLN FDCs and its potential impact on viral resurgence or achieving a cure for HIV infection.

## Introduction

**Figure 1:**
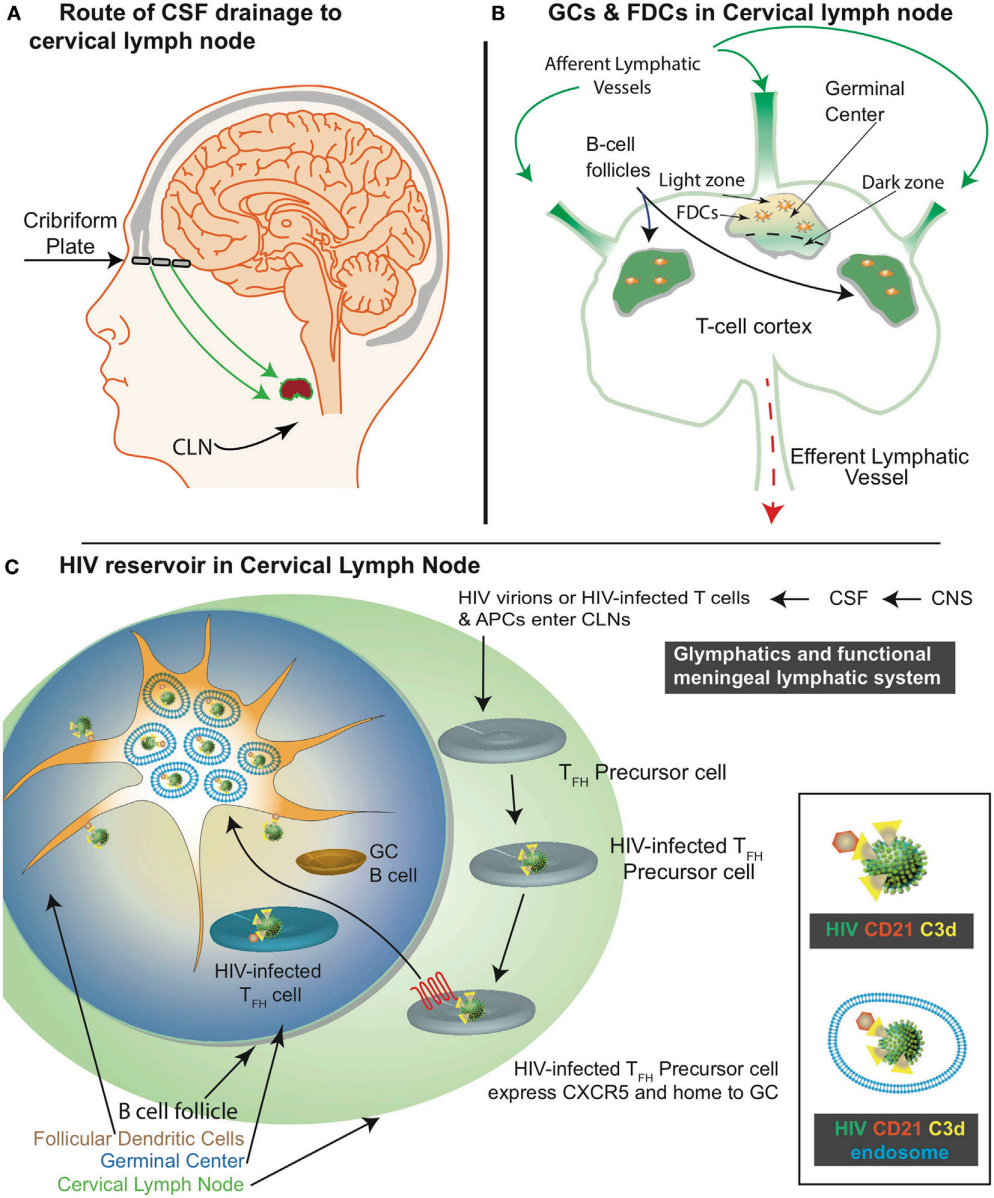
Schematic representation of the central nervous system (CNS)-associated meningeal lymphatic system and the human immunodeficiency virus (HIV) reservoir in the cervical lymph nodes (CLNs). **(A)** The functional meningeal lymphatic vessels drain cerebrospinal fluid (CSF). T cells and antigen-presenting cells migrate with the CSF along the nasal lymphatic pathways through the cribriform plate to access the CLNs. **(B)** CSF enters the CLN *via* the afferent lymphatic vessel and exits through the efferent lymphatic vessels. Germinal center (GC) is located within the B-cell follicle. The follicular dendritic cells (FDCs) are located within the light zone of the GC. **(C)** Within the CLNs, HIV infects T follicular helper precursor cells, which subsequently express CXCR5 and migrate to the light zone. As depicted in the inset, CD21 interacts with C3d on HIV surface. This interaction results in HIV acquisition by the FDCs. Majority of the FDC associated HIV cycles through the endosomal compartment.

The cervical lymph nodes (CLNs) are a group of lymph nodes (LNs) in the neck region that are located adjacent to the cervical region of the spinal cord and in close proximity to the sternocleidomastoid muscle. Depending on the location of the CLNs, they may be classified as (a) superficial anterior CLNs, (b) superficial posterior CLNs, (c) superior deep CLNs, or (d) inferior deep CLNs. The glymphatics and meningeal lymphatics system is a functional waste pathway in the vertebrate central nervous system (CNS) (1, 2). The glymphatics and meningeal lymphatic system connects the CNS with the CLNs (3–10). More importantly, T cells and antigen-presenting cells (APCs) migrate along with the cerebrospinal fluid (CSF) as it drains along the nasal lymphatic path through the cribriform plate and eventually access the CLNs (Figure 1A) (3). Within the LN, there is a network of stromal cells that includes the follicular dendritic cells (FDCs). FDCs were first identified as “antigen retaining reticular cells” (11). Subsequently, FDCs have been recognized for their unique ability to retain antigens for a prolonged duration (12). This property of FDCs is critical for several immune functions, including germinal center (GC) formation and long-term immune memory. FDCs develop from perivascular precursors of stromal cell origin, which are seeded throughout the body. Their maturation requires lymphotoxin alpha and tumor necrosis factor alpha (TNF-alpha) signaling *via* B cells (13). FDCs are found within the B-cell follicles (BCFs) where GCs develop as a result of a T cell-dependent antibody response (14). As the BCFs mature within the GCs, FDCs migrate into the light zone (Figure 1B).

Antigen acquisition, processing, and retention by FDCs impact the immune response. FDCs retain antigens for months to years (15, 16). However, there is inadequate experimental data demonstrating prolonged antigen retention by FDCs (17). In fact, most predictions are extrapolations based on decay rates. In addition to prolonged antigen retention, FDCs can also similarly retain human immunodeficiency virus (HIV)-1 (Figure 1C) (18). The FDC microenvironment is highly favorable for HIV infection (17). There is evidence in support of combined antiretroviral therapy (cART)-mediated viral clearance (19). Of note, there is a study (20) that conflicts this observation. As such, further investigations are necessary to understand if cART diminishes FDC-associated viral reservoir. Nonetheless, FDCs are considered a lymphoid tissue viral reservoir responsible for residual ongoing viremia (21) as well as, a source of viral resurgence upon cessation of cART (22). Of note, HIV retained by FDCs represents a divergent viral archive (23). The CLN FDCs like FDCs within the peripheral LNs also constitute a HIV/simian immunodeficiency virus (SIV) reservoir. In this review, we discuss how CLNs acquire, accumulate, and transmit HIV. In addition, we present some recent advances in FDC-related HIV research (Table 1).

**Table 1:** Advances in follicular dendritic cell (FDC)-related human immunodeficiency virus (HIV) research.

Major findings	Reference
1. Simian immunodeficiency virus (SIV) accumulation in rhesus macaque cervical lymph node (CLN) FDCs and transmission to T follicular helper (T_FH_)	(24)

2. Enrichment of SIV DNA in CTLA-4 + PD-1-memory cells in lymph nodes	(25)

3. Engineering unselected CD8 T cells to express CXCR5 directs them into viral sanctuaries	(26)

4. Identification of a specialized group of CXCR5 expressing cytotoxic T cells that selectively entered B cell follicles and eradicated infected T_FH_ cells and B cells	(27)

5. T_FH_ are a source of replication competent HIV during latency	(28)

6. HIV-exposed FDCs show an increased production of inflammatory cytokines	(29)

7. RNAscope- and DNAscope-based characterization of HIV/SIV lymphoid reservoir	(30)

8. Combined antiretroviral therapy (cART) interruption results in widespread resurgence of rebounding/founder HIV variants	(22)

9. Productive SIV infection is restricted to CD4 + T_FH_ cells in Elite controller macaques and not typical progressors	(31)

10. Trafficking of conventional DCs into germinal center (GC) of CLNs	(32)

11. SIV-infected GC T_FH_ derived from T_FH_ precursor cell subsets	(33)

12. Persistent viral replication in lymphoid tissue due to suboptimal drug penetration	(34)

13. FDCs as a source of low-level viremia	(21)

14. FDCs increase HIV transcription and production by a soluble tumor necrosis factor-alpha-mediated mechanism	(35)

15. FDC-trapped virus was replication competent and demonstrated greater genetic diversity than that of virus found in most other tissues and cells	(23)

16. Anti-CD21 mABs decreases HIV trapping by lymph node cells	(36)

17. Species-specific colocalization of osteopontin with the FDC network in lymphatic tissues in HIV-1 and simian immunodeficiency virus infections	(37)

18. FDC–virus interactions stabilize the virus particle, thus contributing to the maintenance of infectivity	(38)

19. FDCs serve as a reservoir of infectious virus and render surrounding GC T cells highly susceptible to infection with X4 isolates of HIV-1	(39)

20. FDC microenvironment is highly conducive to active HIV infection	(17)

21. FDC-associated virus accumulates soon after infection and cART does not diminish the FDC HIV reservoir	(20)

22. HIV-1 binds to B cells with CD21 receptor	(40)

23. FDCs accumulate HIV for a prolonged duration	(18)

24. FDCs-associated HIV is rapidly cleared with potent antiretroviral therapy	(19)

## CNS and CLN FDCs are Important Components of HIV Neuro-Immunopathogenesis

Human immunodeficiency virus neuroinvasion occurs very early during infection (41, 42) with transmigrating infected monocytes/macrophages (43) and CD4+ T cells (42). In SIV/SHIV macaque models, SIV neuroinvasion occurs within a few days to weeks (44). HIV cannot be eliminated from the CNS as infected monocytes or microglia have a long lifespan and low turnover (45). These monocytes and microglia within the CNS support latent HIV infection (46–49). Since, there is suboptimal penetration of cART (50, 51) across the blood–brain barrier (BBB) resulting in establishment of reservoir within the CNS.

Besides the CNS, HIV persists within the LNs, spleen, gut-associated lymphoid tissue, reproductive organs, and lungs (52, 53). LNs are a known reservoir of persistent HIV/SIV viral infection under suppressive cART (34, 54–58). Several unique characteristics of the LNs contribute to the ability of HIV to persist in this tissue. For example, LN tissue has a slower decay rate than in the peripheral blood (34). Additionally, the LN follicles contain FDCs that capture HIV virions on their cell surface in immune complexes (24). FDCs in the peripheral LNs have also been characterized as another viral reservoir site (21, 35, 59). Importantly, HIV-susceptible T follicular helper (T_FH_) cells are located in close proximity to FDCs, which within peripheral LNs have been shown to trap virions in their native non-degraded state for months to years (60–63) with a half-life of approximately 2–3 months (23, 64). While FDC-trapped virus does not replicate or evolve; however, it can infect nearby trafficking cells (23, 24). Even during cART, replicating virus persists and replenishes trapped stores of HIV (22, 54).

Until the description of glymphatics and the functional meningeal lymphatic system, CNS was considered to be immune-privileged (5). With the elucidation of structural and functional features of this CNS-associated lymphatic system (2, 3, 6), it is now well established that the CNS undergoes constant immune sur-veillance in the meningeal compartment. The meningeal lymphatic system, along with glymphatics presents a unique connection between the CNS and CLNs. HIV may pass with CSF as virions, infected T cells, or APCs through the cribriform plate along the nasal lymphatic pathway and access the CLNs. Lymph entering the CLNs through the afferent lymphatics is channeled through the subscapular sinus into the medulla. The fibroblastic reticular cell (FRC) conduits access afferent lymph and traverse BCFs, where they intersect FDCs. FRC conduits continue into the cortex where they end at high endothelial vessels or the medulla (65).

Recent focus directed at better understanding of the meningeal lymphatic system has tremendously enhanced our understanding of immune surveillance in the CNS (2–9). Lymphatic vessels were first identified in the dura mater of rats (7). In some studies of the meningeal lymphatics (3), the system has been described as part of the CNS, while others have drawn opposing conclusions (9). This is not surprising since lymphatic vessels are component of the surrounding connective tissue that is included in the CNS. However, lymphatic vessels can absorb CSF from adjacent subarachnoid space and brain interstitial fluid *via* the glymphatics. Further detailed investigations are required to fully understand the functionality of CSF drainage, and how it might impact HIV accumulation within CLNs (1).

Circulating conventional DCs (cDCs) are known to traffic into the CNS in response to neuroinflammation (66–73) during HIV/SIV infection (74). Within CNS, cDCs act as both “*carrier and bearer*” of HIV and contribute both to neuropathology as well as CNS reservoir. Recent studies suggest that cDCs may capture HIV within the CNS and deliver it to different compartments of CLNs including FDCs (32, 60, 61, 75). The CLN FDCs would create a viral repository where virus can remain bound for prolonged duration (63). The immune cell retrograde transport studies (3, 6, 76, 77) provide clues that cDCs upon encountering HIV virions within the brain would migrate along the meningeal lymphatic vessels to draining LNs (CLNs, near the brain stem) *via* glymphatics delivering HIV particles to different CLN compartments including FDCs as shown for peripheral LNs (60, 61, 75). However, CLNs are the major site for systemic activation of CNS-specific T cells. They receive input from the CNS in the form of antigens and cDCs (78). Within CLNs, HIV viral particles may be transmitted to CD4+ T cells or trapped on the FDC network, thereby stabilizing and protecting HIV and creating a long-term reservoir of infectious HIV (21, 34, 38, 54). In addition, FDCs activate CD4+ T cells within GCs and increase virus production in these cells even in the presence of cART (35, 39, 79–81). Assessing the involvement of CLNs in HIV neuropathogenesis is timely, given our recent advances in understanding of the functional meningeal lymphatic system (3). Of note, additional mechanistic studies are required to determine if the CLN FDC reservoir is an archive of CNS egressing virus.

## Key Cellular Players in the CLN

Cervical lymph nodes like other LNs play a central role in the development of adaptive immunity against pathogens and, particularly, the generation of antigen-specific B cell responses in specialized areas within GCs (82). Very early in the HIV epidemic, LN pathology was recognized as an important consequence of HIV infection since the beginning of the epidemic. Studies focused on lymphoid tissue architecture during HIV/SIV infection have highlighted the key role of the ln in the disease pathogenesis. The LN environment is unique for viral evolution, primarily because of the relative exclusion of HIV-specific CD8 T cells (83). In a subsequent study, SIV-specific CD8 T cells in GC and non-GC regions were quantitated (84). Therefore, further investigations are necessary to understand the biology of immune cells in HIV-infected LNs and their critical role in achieving complete viral eradication.

Follicular dendritic cells are a subset of DCs that are of mesen-chymal origin and essential for GC formation and production of various types of antibodies (16). They reside in secondary lymphoid tissues such as spleen, tonsils, LNs, and follicles that appear at extranodal sites (85). GCs of secondary lymphoid tissues are composed of several types of immune cells, such as activated B cells, T_FH_ cells, and FDCs. FDCs interact with their GC counterparts. In the GC microenvironment, activated B cells communicate with FDCs by interacting with an antigen on their surface and then present this antigen to T_FH_ cells. FDCs can select for B cells to re-enter the GC or exit with the help of T_FH_ cells (15). FDCs have a unique ability to retain immune complexes on their dendritic processes. These immune complexes consist of antigen–antibody complexes and complement (86), which can retain infectious virions for several months even in the presence of neutralizing antibodies or under cART (54). FDCs interact with T_FH_ cells in GCs, and these cells serve as a reservoir of infectious virus. Surrounding GC, T cells become highly susceptible to infection with HIV X4 isolates (39). HIV production increases to twofold when viral particles are transferred from FDCs to susceptible CD4+ T cells (35). FDCs can secrete inflammatory cytokines (29) including TNF alpha and thereby contribute to enhanced transcription in the LNs (35).

The underlying mechanisms of HIV/SIV FDC reservoirs remain unclear and require further studies. FDC trapped HIV virions in human lymphoid tissues remain infectious (56). In murine FDCs, HIV virions in immune complexes remained infectious *ex vivo* for up to 9 months after being captured by FDCs (54). This characteristic of the FDCs is particularly interesting because most of the identified reservoirs of persistent virus are found in the integrated pro-viral stage of the HIV replication cycle. It is important to note that current approaches to eliminate persistent HIV have largely focused on elimination of HIV pro-viral DNA. The HIV/SIV lymphoid reservoir has been well characterized utilizing RNAscope and DNAscope methodologies (30). However, the CLN FDC HIV reservoir has only recently been partially characterized (24) and requires detailed investigation (Table 1).

T follicular helper are a subset of CD4 T lymphocytes (87) that play a key role in B-cell differentiation. T_FH_ cells assist B cells in the production of antigen-specific antibodies and are essential for memory B cell activation, survival, and differentiation. Even under conditions of durable control, such as in elite controller macaques, T_FH_ cells contribute significantly to ongoing viral replication and production, and are the single CD4 subset in the LN’s most highly enriched in SIV (31). During HIV infection, cellular interactions between FDCs, GC B-cells, and T_FH_ cells result in reservoir establishment. T_FH_-associated replication competent virus may be the source of resurgent HIV after cART interruption or failure. As such, T_FH_ are increasingly recognized as another major CLN-associated reservoir of HIV infection (88–90). However, mechanisms by which these cells get infected remain unclear. T_FH_ express very little CCR5 and in macaque studies, it has been shown that T_FH_ lacking CCR5 cells can be infected *in vivo* with CCR5-tropic SIV (91, 92). Infection of the T_FH_ population by CCR-5 tropic viruses appears to be the result of infection of the pre-T_FH_ cells that express CCR-5 (93).

In cART-naïve as well as treated individuals, T_FH_ and GC B cells are elevated (94). In addition, there is a direct correlation of T_FH_ and GC B cells with the activated T-cell population in the LNs (95). In absence of cART, during chronic HIV infection, viral replication is concentrated in secondary lymphoid follicles (SLF). T_FH_ cells have been shown to be highly permissive to HIV within SLF and are the source of replication competent HIV during latency (28). HIV vaccines are not strong inducers of neutralizing antibodies. However, in one of the recently described study, rhesus macaques were immunized with HIV envelope glycoprotein trimer, and there was a substantial production of HIV neutralizing antibodies (96). The high antibody titers had a strong correlation to GC B cells and T_FH_ (96). These observations underscore the need to study more details of LNs, since previous HIV reservoir studies have frequently focused primarily on the peripheral blood.

Follicular regulatory T (TFR) cells are another subset of T cells in SLF (97, 98). TFR share some phenotypic characteristics with the T_FH_ cells. Importantly, both T_FH_ and TFR are permissive to HIV infection (99). However, TFR express greater levels of CCR5 and CD4 as compared with the T_FH_ cells. They also support higher frequency of viral replication. Expression of Ki67, a marker of proliferative capacity appears to correlate with viral replication in these cells. As such, TFR differ from T_FH_ in their susceptibility to R5 HIV infection (99). Furthermore, it has recently been shown that natural killer (NK) cells migrate into the follicles of secondary LNs. The role of NK cells in LNs is not clear. However, a particular study in African green monkeys demonstrated that entry and persistence of NK cells in LNs was IL-15 dependent, as depletion of IL-15 resulted in an increase in viral replication. These data suggest a key role for NK cells in the establishment and maintenance of this viral reservoir (100).

## Eradication of FDC Viral Reservoir

A significant challenge to HIV eradication is the elimination of viral reservoirs in GC T_FH_ cells. GCs are considered to represent an immune privileged site within the LN where antiviral CD8+ T cells are primarily excluded (83, 84). However, unselected CD8+ T cells engineered to express CXCR5 (C-X-C chemokine receptor type 5, a chemokine receptor required for homing to GCs) direct them to GCs (26). CXCR5 expressing cytotoxic T cells are able to selectively enter BCFs and eradicate infected T_FH_ and B cells (27). A population of SIV-specific CD8+ T cells expressed CXCR5 and expanded in LNs following pathogenic SIV infection in a cohort of vaccinated macaques (101). Animals that exhibited greater control of SIV replication had a greater expansion of these cells. The increase in CXCR5+ CD8 T cells was associated with the presence of higher frequencies of SIV-specific CD8 T cells in the GC (101). Thus, CXCR5+ CD8 T cells represent a unique subset of antiviral CD8+ T cells that expand in LNs during chronic SIV infection and may play a significant role in the control of pathogenic SIV infection (101) (Table 1).

An important milestone in purging the FDC reservoir was demonstrated by utilization of soluble complement receptor 2 or CD21 (102). CD21 is necessary for HIV interaction with FDCs and B-cells (40) as interaction of HIV with FDC stabilizes the virus (38). Despite the stabilized interaction, Heesters and co-workers were able to purge the FDCs of HIV virions by utilizing a soluble form of CD21 (102). Thus, intersecting CD21:C3d interactions significantly reduced recycling of virions through the endosomal compartment. In addition, viral transmission to T_FH_ was diminished in *in vitro* studies (102). In an alternate approach to purge FDC HIV reservoir, monoclonal antibodies targeting CD21 were utilized (36). Thus, blocking CD21 interactions appears to be a potential strategy for purging the FDC HIV reservoir.

## Future Perspectives

Profound and durable suppression of HIV by cART represents a major accomplishment in HIV/AIDS research (103, 104). However, HIV persists in patients despite long-term administration of cART (55). Withdrawing cART invariably results in viral rebound (105, 106). One of the major challenges with cART is to maintain virologic control. Two types of research strategies have been utilized in HIV cure research. Eradication of replication-competent HIV is considered as a “classic cure” and the best example is Timothy Brown, also known as the Berlin patient (107). Timothy Brown received a stem cell transplant from a donor that was homozygous for delta32 mutation in the CCR5 gene (107). On the other hand, in a “functional cure,” viral rebound after cessation of cART is controlled without eradication of HIV. Such functional cure was demonstrated in SIV-infected rhesus macaques with α4β7 monoclonal antibody (108). Even after cART withdrawal, sustained virologic control was maintained with passive administration of α4β7 monoclonal antibody (108). However, mechanisms underlying this sustained virologic suppression remain to be elucidated. Other HIV cure strategies include (a) latency-reversing agents (e.g., anti-CD3, Bryostatin, IL-7, Romidepsin, TLR-7 agonists, Valproic acid), (b) immuno-toxic therapy with bi-functional antibodies, and (c) precise excision of HIV genomes by CRISPR/Cas9 gene editing in mice (109–111). It needs to be determined if such cure strategies can successfully purge the CNS and the LN HIV reservoirs based on our current understanding of the functional meningeal system, CSF outflow (1), and viral acquisition by FDCs (Figures 1A–C) (24). Lifetime cART is associated with toxicity, residual chronic inflammation, and the accelerated onset of comorbidities associated with aging. Therefore, optimizing other cure strategies in combination with cART will be critical to reducing cART-associated complications and the overall viral burden.

## Author Contributions

RD: wrote the review article. PJ and SB: edited the review article.

## Conflict of Interest Statement

The authors declare that the research was conducted in the absence of any commercial or financial relationships that could be construed as a potential conflict of interest. The reviewer SB and handling Editor declared their shared affiliation.
